# A pipeline for developing deep learning prognostic prediction models in cardiac magnetic resonance image analysis

**DOI:** 10.1093/ehjdh/ztaf101

**Published:** 2025-08-28

**Authors:** Mattia Corianò, Corrado Lanera, Pier Giorgio Masci, Gianluca Pontone, Martina Perazzolo Marra, Dario Gregori, Francesco Tona

**Affiliations:** Cardiology Unit, Department of Cardiac, Thoracic, Vascular Sciences and Public Health, University of Padua, Via Nicolò Giustiniani, 35121 Padova PD, Italy; Department of Cardiac Thoracic Vascular Sciences and Public Health, UBEP, Padova, Italy; School of Biomedical Engineering and Imaging Sciences, Faculty of Life Sciences and Medicine, King’s College London, London, UK; Department of Perioperative Cardiology and Cardiovascular Imaging, Centro Cardiologico Monzino IRCCS, Milan, Italy; Department of Biomedical, Surgical and Dental Sciences, University of Milan, Milan, Italy; Cardiology Unit, Department of Cardiac, Thoracic, Vascular Sciences and Public Health, University of Padua, Via Nicolò Giustiniani, 35121 Padova PD, Italy; Department of Cardiac Thoracic Vascular Sciences and Public Health, UBEP, Padova, Italy; Cardiology Unit, Department of Cardiac, Thoracic, Vascular Sciences and Public Health, University of Padua, Via Nicolò Giustiniani, 35121 Padova PD, Italy

**Keywords:** Artificial intelligence, Deep learning, Sudden cardiac death, Cardiomyopathy, Prediction model

## Abstract

Patients and healthcare professionals require clinical prediction models to accurately guide healthcare decisions, although an awareness of the limitations of regression-based models has recently increased. Deep learning (DL) has emerged as a promising alternative to traditional regression-based models, due to its ability to effectively analyse heterogeneous types of data, ranging from numerical variables to medical images.

Building a DL model presents various challenges, including conceptualizing the clinical problem, selecting appropriate variables and model architecture, and providing explainability. We propose a four-step pipeline for developing DL-based prediction models for cardiac magnetic resonance image analysis. This framework aims to support researchers in exploring DL application across the broad spectrum of cardiology, with a specific focus on advancement in arrhythmic risk prediction.

The field of cardiomyopathy faces challenges when assessing arrhythmic risk due to the low accuracy of the current prediction models. Research efforts have focused on developing DL models able to predict major arrhythmic events in dilated cardiomyopathy. While the initial results are promising, further tests are needed before translating these models into clinical practice.

## Graphical Abstract

**Graphical Abstract:**
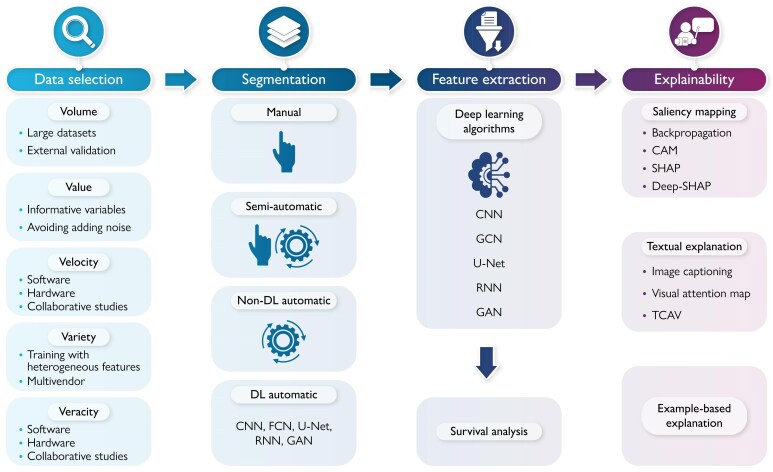
The figure summarizes the four steps presented in the manuscript. The first step involves selecting the data according to the ‘five V’ principle. The second step involves deciding whether and how to apply a segmentation model during the pre-processing phase, in order to isolate only the relevant regions of interest and reduce noise and potential errors. The third step is feature extraction, where the region of interest is processed to predict a pre-specified outcome. The final step is to ensure the model's explainability, avoiding the ‘black box’ effect and making the model more trustworthy.

## Introduction

Forecasting prognosis is constantly required in daily clinical practice for informed decision-making in agreement with patients. Prediction models are classically employed to estimate the probability of a certain outcome occurring. In cardiovascular science, >600 models have been proposed to assess cardiovascular risk, ranging from sudden cardiac arrest,^1^ to general population and sex-specific models.^2,3^ Despite this sheer number of models, few are actually used in clinical practice given that most perform poorly particularly for outcome prediction and effective therapeutic strategies. The sudden cardiac death (SCD) risk prediction in patients affected by cardiomyopathy represents a valid example of this challenge, given that inaccuracy in model prediction can affect patients’ survival and quality of life.^4^

The spread of artificial intelligence (AI), and in particular of deep learning (DL), has gained enthusiasm about the possibility of increased accuracy of prediction models. However, this initial excitement has been tempered by several challenges in the field, such as inconsistent methodologies in model development, inadequate testing of model performance, and limited explainability and interpretability of results. The awareness of these limits led to the writing of the ‘Transparent Reporting of a Multivariable Prediction Model for Individual Prognosis or Diagnosis Based on Artificial Intelligence’ (TRIPOD-AI).^5^ In this document, the authors suggest three main aspects to be considered when developing an AI model. Firstly, the model should solve a target medical problem and fit into the existing clinical workflow. Secondly, data should be large enough to represent the targeted population, and the process should be reliable and replicable. Thirdly, the model should be rigorously tested and the results correctly reported and explainable. Considering this, TRIPOD-AI suggests general principles for the project management of the research, although a practical approach of the methodology to be used for the model's development is not present.

Here, we propose a pipeline for developing a DL model for risk prediction able to analyse cardiac magnetic resonance (CMR) images. In the first part, we introduce prognostic prediction models, emphasizing how DL promises high accuracy in risk prediction compared with regression-based models and standard machine learning (ML) models. Then, we propose a four-step approach for model's building, dwelling on pioneering studies of DL for arrhythmic risk prediction in cardiomyopathies. The suggested approach included data selection, cardiac segmentation, feature extraction, and explainability. This framework aims to support researchers in exploring DL application across the broad spectrum of cardiology.

## Prediction models: from regression to deep learning

Prediction models are part of the everyday clinical practice in cardiology, and their importance has drastically increased in the field of arrhythmic risk prediction. With the availability of always new treatments, sometimes expensive, an accurate outcome prediction gives healthcare professionals and patients more information about risks, allowing them to choose a personalized treatment strategy and to better allocate resources. Prediction models can be classified into two major categories: diagnostic and prognostic. A diagnostic prediction model estimates an individual's probability of a specific health condition being currently present, while a prognostic prediction model estimates the probability of developing a specific health outcome over a specific time period.^6^ Regression models have classically been employed to develop prediction models, where logistic regression analysis is commonly used for the prediction of binary events and Cox regression is adopted for time-to-event data.^7^ Although regression-based prediction models are easily understandable and applicable in clinical practice, their accuracy in correctly discriminating patients at high or low risk of major events has been widely criticized. The limits of regression models were extensively reviewed by Myerburg and Goldberger.^8^ In their work, the main limitations highlighted were the use of a specific cut-off for continuous variables and the assumption of a linear relationship between clinical variables under investigation and outcome. In this scenario, AI has gained enthusiasm in recent years as a promising alternative to traditional regression-based models. In fact, ML algorithms can handle a wide range of data and find more complex relationships between risk factors and outcomes. Prediction models based on simple ML algorithms [i.e. nearest neighbours, decision trees, and neural networks (NNs)] were tested in different settings, from cardiovascular prevention in the general population to sudden cardiac death (SCD) prevention in cardiomyopathies, using data variables as input.^4^ Even though the accuracy of these models was slightly superior compared with regression-based models, the overall performance and the methodology of building and testing the models were not convincing enough to translate them into clinical practice.^9,10^

In the last years, new opportunities to overcome these limitations have emerged with the spread of DL. The term DL refers to a subfield of ML that employs artificial NNs containing multiple layers to form a meaningful representation of data, though at a higher computational cost. The recent advancement of computational power and mathematical algorithms underpinning AI, the decreased cost of graphic processing units (GPUs), and the increased number of publicly available datasets allowed DL to thrive.^11,12^ DL has been shown to outperform ML strategies in various areas, especially when there is a large volume of data to be analysed.^13–15^ Considering the unmet needs still present, cardiovascular science represents a fertile ground for testing DL algorithms to pursue precision medicine, with numerous applications previously reported across different fields of cardiology.^16^ As an example, DL has been used to detect arrhythmias from electrocardiograms (ECG) or wearable devices,^17–19^ classify diseases,^20,21^ or segment images.^22^ Moreover, DL has been applied across the spectrum of CMR, including image plan prescription,^23^ accelerated image acquisition and reconstruction,^24^ cavity segmentation,^25,26^ report generation,^27^ and risk prediction.^11,28^

Although these applications seem promising, building a DL prediction model poses many challenges. Factors such as the type of images to include, segmentation methods, feature extraction, and explainability all need careful consideration. Currently, there is no standardized pipeline to follow; however, common approaches can be identified across studies. In the following paragraphs, we propose a possible pipeline for the development of a DL prognostic model based on CMR images, comparing various approaches employed by different researchers in the field of arrhythmic risk prediction. The suggested pipeline is shown in the *Graphical Abstract* and is divided into four main parts: data selection, image segmentation, feature analysis, and feature explainability.

## Designing deep learning prognostic models from CMR data

### Input data selection

Data quality is crucial for developing a highly performing DL model. A common motto in data science cites ‘garbage in, garbage out’, meaning that a low quality of input data surely compromises its accuracy. Ideally, a DL model should be built using ‘big data’, intended as a database characterized by the ‘five V’: volume (large dataset), velocity (fast and efficient), variety (originated from different sources and containing enough variance), veracity (containing accurate and trustworthy data), and value (containing information needed to address a given question).^12^

While managing volume, velocity, and value requires adherence to general principles, ensuring veracity and variety necessitates practical steps that depend on the data type.

#### Volume, velocity, and value of data

Although it was historically meant that a large dataset is required to train a DL model, no formal sample size criteria exist. Some authors suggested *a priori* sample size estimation similar to a regression-based model, while others preferred *a posteriori* calculation.^29–32^ Both methods have many limitations, and a recently widely accepted position is that the sample size should be large enough to ensure that predictive performance measures can be estimated with sufficient reliability and scalability.^10^ With this assumption, the attention has moved towards the external validation dataset, which should be representative of the target population and sufficiently large to properly test the real performance of the model.

Concerning the value of data, variables and images should be carefully chosen according to the preexisting literature evidence, trying to include the major number of informative variables and, at the same time, to avoid adding noise to the model (i.e. low predictive effects of predictors and features).^33,34^

A different consideration pertains to data velocity. The speed of training of a DL model depends significantly on the quality of software and hardware architectures. While DL packages are mostly uniformly open-source, professional-quality GPUs remain relatively expensive. Multicentre collaborative studies can alleviate these costs by providing access to more powerful computing resources and sharing the expense of computation that individual institutions might not afford independently. This collaborative approach not only accelerates model training but also promotes the pooling of diverse datasets, thereby enhancing the generalizability and robustness of the DL models developed.

#### Variety and veracity of data and images

Contrast sensitivity, spatial resolution, noise, and artefacts are features of CMR images, and their presence depends on patient characteristics, vendor machine, and scan protocol. These aspects should be considered when a large dataset for training a DL model is analysed.

The impact of training data diversity on DL model performance in CMR has been increasingly studied, with recent research highlighting its critical role in improving robustness and generalizability. Recent studies have explored the role of training data diversity in DL models for CMR. Hoh *et al*.^35^ demonstrated how a wide variety of input data improves the performance of a DL model for late gadolinium enhancement (LGE) CMR image analysis, showing that training on multiple resolutions reduced resolution dependency. Scannell *et al*.^36^ demonstrated that models trained on homogeneous datasets often underperform when applied to data from varied sources. They found that including images from different centres, vendors, and patient populations significantly improves model robustness and generalization. Additionally, Puyol-Antón *et al*.^37^ highlighted that DL models trained on imbalanced datasets exhibited biases across different demographic groups, further reinforcing the need for diverse training data to ensure fairness and accuracy.

The findings of these studies enhance how input images should be chosen for developing a DL model. Specifically, the variety of the data in the training dataset should reflect the variety of the data in the test set. In other words, if a DL model is meant to be used only in a specific setting of clinical images, with standardized image parameters (e.g. constant spatial resolution, vendor machine, and magnetic fields), a model trained with the same image features as the test set will perform better than a model trained on multiple image variations. On the contrary, a model intended to analyse images with different features will perform better if trained with a varied dataset. Considering the high heterogeneity of data in cardiovascular imaging, the latter approach is preferable and would allow for a wider use of the DL model.

### Cardiac segmentation

Once the quantity and type of CMR images to be used as input variables for a DL model have been determined, the subsequent critical task to handle is cardiac segmentation. Image segmentation is classically defined as a process of partitioning an image into a number of semantically and anatomically meaningful regions, based on which quantitative measures can be extracted.^26^ This partitioning involves classifying pixels within an image of an organ or substructure based on their specific features.^38–40^ After segmentation, the process of feature extraction, selection, and classification may occur. In classical cardiac image analysis, segmentation is a challenging task that significantly influences the accurate interpretation of the images.

The segmentation approach of CMR images can be defined as manual, semi-automatic, and automatic.^22^ The first requires experts to analyse each slice of 2D or 3D images and annotate regions of interest. The advantage of manual segmentation is that it utilizes expert knowledge to comprise the ground truth. On the other hand, it is laborious, time-consuming, and prone to inter-observer variability. Semi-automatic segmentation relies on an automatic framework, followed by manual review. While this method allows to save time in labelling, the major disadvantages are its reliance on manual initialization and, therefore, the inter-observer variability. Fully automatic segmentation is more time-efficient and reliable as it requires no manual input, though susceptible to inaccuracies when handling artefacts or non-standard windows.

#### Deep learning for cardiac segmentation

There is no clear consensus on the type of segmentation to apply to CMR images when building a DL prediction model. Ideally, a DL model should be trained with trustworthy data, making a manual segmentation the most suitable way to remove artefacts and label critical parts of images like basal and apical slices. Conversely, employing a manual or semi-automatic segmentation on a wide dataset is unrealistic due to the extremely high time required to complete the task. Therefore, in previous research, a fully automated segmentation strategy was applied to preprocess CMR images.^41–46^

**Figure 1:**
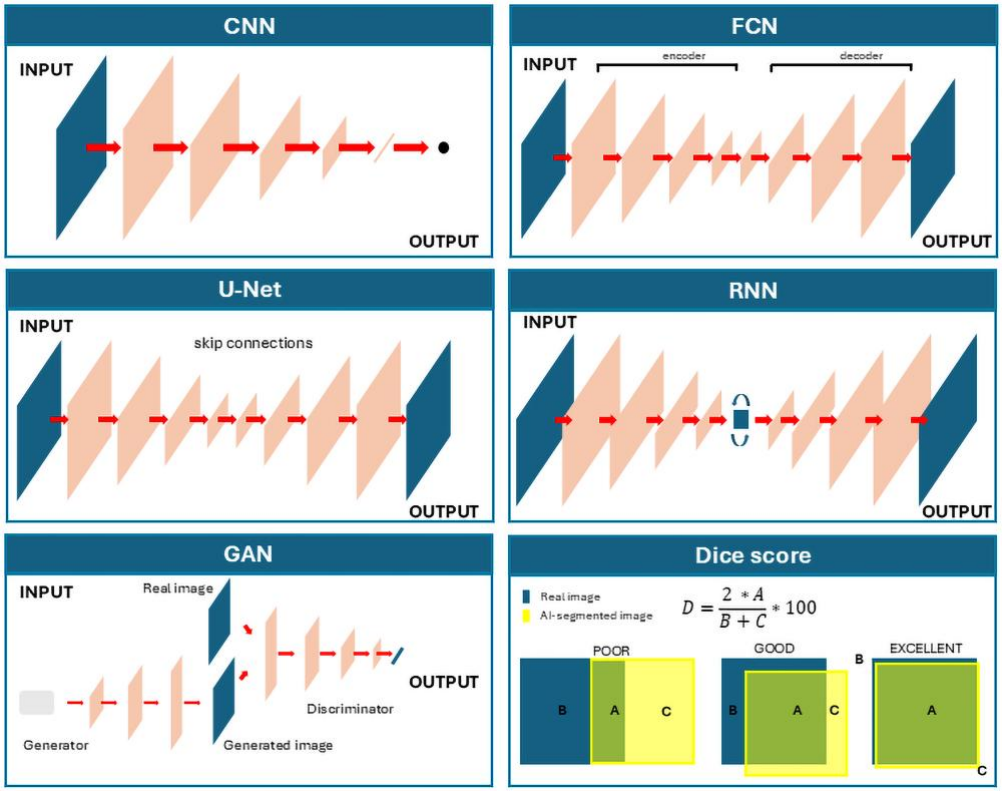
The main type of deep learning models used for cardiac image segmentation. *CNN (top left):* a convolutional neural network learns hierarchical features through a stack of convolution and pooling operations. The feature maps are then flattened and reduced into a vector. The final vector can represent a set of classes (for image classification), coordinates of a bounding box (for object localization), or a real value (for regression tasks). *FCN (top right):* a fully convolutional neural network learns features during the encoding process and recovers spatial dimensions during the decoding process. It produces probabilistic maps to predict regions of interest. The final segmentation map is obtained by assigning each pixel the class with the highest probability. *U-Net (centre left):* U-Net is a variant of FCN that adds skip connections to aggregate feature maps through concatenation and convolutions. *RNN (centre right):* a recurrent neural network utilizes knowledge learned from the past to make future predictions. *GAN (bottom left):* generative adversarial networks can create new image examples after learning the data distribution of real data. As shown in the figure, a GAN consists of a generator network and a discriminator network that compete against each other during training. The generator produces fake images, and the discriminator tries to distinguish between real and fake images. *Dice score (bottom right):* the Dice score is the most commonly used metric to evaluate segmentation performance. It is calculated as the ratio of twice the area of correct matching between the ground truth and the segmented image to the sum of the two areas. A value of 1 indicates perfect matching, while a value of 0 indicates no matching.

Both traditional and DL-based methods can be used for automatic image segmentation; however, DL approaches are particularly known for their high accuracy and robustness to anatomical variations.^40,47^ Overall, DL algorithms for image analysis address two main challenges: analysing images on a pixel-by-pixel basis while preserving the spatial hierarchy of features and reducing dimensionality without losing important information. Different kinds of DL algorithms have been tested in segmentation tasks and compared with the manual segmentation as ground truth. A brief overview of the main types of DL architectures used for segmentation of CMR images is reported below and shown in *Figure 1*. An extensive discussion of this topic can be found elsewhere.^40,47,48^

##### Convolutional neural networks

Convolutional neural networks (CNNs) represent the most common type of deep NN for image analysis. CNNs are built using the concept of convolution, intended as the process of applying a filter (kernel—a small matrix of weights) to an input matrix (i.e. an image) to produce an output matrix (feature map). The functioning of a standard CNN model can be summarized as a three-step process. Firstly, the model learns hierarchical features through convolution and pooling operation creating a spatial feature map. Secondly, the feature maps are subjected to a progressive flattening (i.e. dimension reduction), executed by a stack of functional layers containing convolutional, pooling, and fully connected layers. Thirdly, a final vector is obtained after the functional layers. This resulted vector can be represented in many forms, according to the specific task. In segmentation tasks, it is a coordinate of a bounding box that allows object localization. When applied to image segmentation, the process is done by dividing images into patches, resulting in an inefficiency due to the need to process overlapping patches individually leading to redundancy. Therefore, CNNs are primarily used for object localization to crop the images.^49–51^

##### Fully convolutional neural networks

Fully convolutional neural networks (FCNs) do not contain any fully connected layers and are designed with an encoder–decoder structure. This structure allows them to take input of arbitrary size and produce output of the same size. FCNs use the entire image as input, which the encoder transforms into a high-level feature representation. The decoder then interprets these feature maps and recovers spatial details through upsampling and convolution operations. The output consists of pixel-wise probabilistic maps, where the final segmentation map is obtained by assigning each pixel the class of highest probability. Although the structure is flexible and the output is usable, classic FCNs are limited in their ability to capture detailed, complex information, and some features may be deleted in the encoder.^52^

##### U-networks

U-networks (U-Nets) are a variant of FCNs, which employ skip connection, consisting of concatenations and convolutions, between the encoder and decoder to recover spatial context loss. The U-Net has two main parts. The first part, the encoder, reduces the image size step by step using convolutional layers to capture important features and overall context. The second part, the decoder, gradually increases the image size again using upsampling, refining details to make precise pixel-level predictions. A key feature of U-Net is the skip connections, which transfer information from the encoder to the matching layers in the decoder. This helps the model retain important details and improves accuracy in identifying objects within an image.^53^ This novel structure has become the most popular in the field of medical image segmentation, and several variants of the model have been derived to progress the state of the art based on it. The autoencoder design of U-Net makes it a unique tool for a variety of applications.^25,53–56^

##### Recurrent neural networks

Recurrent neural networks (RNNs) are used for sequential data with a temporal dimension. These NNs propagate information from temporally adjacent slices to maintain inter-slice coherence. Specifically, given a sequence of images, an RNN takes the first image as input, captures the information, memorizes it, and then utilizes it to make predictions for subsequent images.^57^ One of the most widely used architectures in the family of RNNs is long short-term memory (LSTM), which is capable of modelling long-term dependencies and memory.^58^ In the field of CMR, RNNs, particularly LSTM, have been used to analyse sequential data such as cine images.^59,60^

##### Generative adversarial networks

Generative adversarial networks (GANs) are generative models able to model data distribution and create new image examples. Their structure consists of a generator network and a discriminator network. The peculiarity of these models is the training phase, consisting of adversarial training where the generator produces images while the discriminator tries to identify the real ones. Currently, few experiments have been done in the field of cardiac segmentation, though GANs seem very promising for producing more anatomically plausible segmentation maps.^61,62^

#### Choice of DL model for CMR image segmentation

The choice of the model to use for segmentation tasks depends on the analysis planned and the disease-related distinctive feature. At the current state-of-the-art, most of the studies have focused on the segmentation of cardiac chambers, while few works on segmenting abnormal cardiac tissue regions such as myocardial scars have been published.^26^

Overall, the choice of a DL model is based on its performance, which can be evaluated by different types of metrics. A description of all the indices used in cardiac image analysis is out of the scope of this manuscript and can be found elsewhere.^63^ Currently, the most used one is the Dice–Sørensen coefficient score, a volume-based metric that measures the ratio of overlap between two results. It ranges between 0 and 1, assigning a value of 0 when there is a total mismatch and a value of 1 when a perfect matching is present. Many models with good performance have been tested for different applications and are freely available. Segmenting cine images is the most commonly tested task, although mapping sequences and scar detection have also been tested.^26^  *Table 1* reports the models with the highest performance in segmenting cine, LGE, scar, and mapping sequences, whereas different ready-to-use tools are proposed in the ‘Case study’ section. Of note, all the proposed models were trained and tested on different datasets and cannot be compared. Moreover, they were built using data of patients affected by certain conditions, and a similar performance cannot be assured on other cohorts of patients.

**Table 1:** Recent deep learning models for segmentation of cardiac magnetic resonance images

Reference	Year	Stack	Best performance (Dice score at end diastole)	Dataset	Repository
Cine images					
Bai *et al*.^64^	2018	SAx, Lax	LV: 0.94 RV: 0.90	UK Biobank	https://github.com/baiwenjia/ukbb_cardiac
Attar *et al*.^65^	2019	SAx, Lax	LV: 0.93 RV: 0.87	UK Biobank	—
Abdeltawab *et al*.^66^	2020	SAx	LV: 0.94	MICCAI 2019 (56 subjects)	—
Budai *et al*.^67^	2020	SAx	LV: 0.93 RV: 0.87	Multiple datasets (MICCAI, ACDC, etc.)	https://github.com/adamtiger/CPmethod
Dong *et al*.^68^	2020	SAx	LV: 0.95	56 subjects	https://lvquan19.github.io/
Wu *et al*.^69^	2020	SAx	LV: 0.95	45 subjects	—
Cheng *et al*.^70^, Chen *et al*.^71^	2020	SAx	LV: 0.95	ACDC (150 subjects)	https://github.com/c-feng/DirectionalFeature https://github.com/cherise215/CardiacMRSegmentation
Hu *et al*.^72^	2021	SAx, LAx	LV: 0.93 RV: 0.83	UK Biobank	—
Penso *et al*.^73^	2021	SAx	LV: 0.94 RV: 0.91	Single-vendor, single-centre (210 subjects)	https://github.com/Monzino/Ventricle_segmentation
Shen *et al*.^74^	2021	SAx	LV: 0.92 LV: 0.94	ACDC (150 subjects)	https://github.com/dsc936/DenseUnet_for_TPM_segmentation
Xia *et al*.^75^	2022	SAx, LAx	LV: 0.88 RV: 0.85	UK Biobank	—
Shoieb *et al*.^76^	2023	SAx	LV: 0.95 RV: 0.90	Multiple dataset (ACDC, MICCAI, etc.)	—
Mariscal-Harana *et al*.^77^	2023	SAx	LV: 0.94	Multivendor, multicentre, >3000 patients	—
LGE					
Xing *et al*.^78^	2022	SAx	LV scar: 0.69	Single-vendor, single-centre (168 patients)	—
Popescu *et al*.^43^	2022	SAx	LV: 0.93	Multivendor, multicentre (220 subjects)	—
Ghanbari *et al*.^45^	2023	SAx	LV scar: 0.67	Multivendor, multicentre (761 subjects)	—
Zhang *et al*.^79^	2023	LAx	LA: 0.89 LA scar: 0.64	MICCAI 2022 (60 subjects)	https://github.com/level6626/Boundary-focused-nnU-Net
Tavakoli *et al*.^80^	2025	SAx	LV scar: 0.91	Multicentre, multivendor (552 subjects)	https://github.com/NedaTavakoli/ScarNet
Mapping					
Bhatt *et al*.^81^	2022	T1 SAx	LV: 0.81 T1/ECV: 0.87/0.92^a^	Single-centre (100 subjects)	—
Chang *et al*.^82^	2022	T1 SAx	LV: 0.81 T1/ECV: 0.97/0.98^a^	Single-centre (95 subjects)	—
Howard *et al*.^83^	2022	T1 SAx, T2 SAx	LV: 0.97 T1/ECV/T2: 0.97/0.99/0.90^a^	(807 subjects)	https://github.com/jphdotam/T1T2_labeller

Performance is considered for segmentation of LV and RV at the end-diastolic phase of the cardiac cycle. When many algorithms were tested, the one with the highest performance was reported.

ACDC, Automated Cardiac Diagnosis Challenge;^84^ ECV, extracellular volume mapping; LA, left atrium; LAx, long-axis; LGE, late gadolinium enhancement; LV, left ventricle; MICCAI, medical image computing and computer-assisted invention challenge;^85^ RV, right ventricle; SAx, short-axis; T1, T1 mapping; T2, T2 mapping.

^a^Expressed as a correlation coefficient between automatic and manual.

### Feature extraction and survival analysis

After isolating the region of interest, the next step consists of the feature extraction. This process identifies and isolates important patterns or characteristics from raw data that are relevant for a specific task, such as risk prediction. By extracting features, the model focuses only on the most relevant information, making it easier to learn and make predictions. Moreover, feature extraction reduces the amount of data the model needs to process, speeding up training and inference. The process can be performed using different DL architectures, which typically consist of various combinations of the DL structures mentioned in the previous paragraph.

### Explainability

It is well recognized that the more complex an AI architecture is, the less explainable it becomes. This lack of explainability is especially concerning in high-stakes decision-making areas, such as risk prediction and image analysis. In fact, interpretable DL models are crucial for the trust of patients and clinicians, as well as for legal reasons.^86^

Often, the term explainability is used interchangeably with interpretability, although both terms have different meanings. Interpretability refers to the ability to present in understandable terms to a human and is mainly connected with the intuition behind the outputs of a model.^87^ On the other hand, explainability is associated with the logic inside an AI algorithm and refers to a deeper understanding of what the model learns during each process.^88^ Therefore, interpretability does not entail explainability, and many authors support that the former alone is insufficient while the presence of explainability is paramount.^86,89,90^

To overcome this situation and to simplify the adoption of AI models in clinical practice, research interest in the field of explainable AI has increased in recent years.^89^ In regard to image analysis, different approaches have been proposed to explain the result of a DL model. In the following paragraphs, we report a brief overview of the main algorithms used to better understand the DL model for CMR image analysis. Of note, an extended description of the explainable AI can be found elsewhere.^86,90,91^

#### Classification of explainable AI

Explainable AI techniques can be classified at different levels. The first distinction is between a model-based explanation vs. a *post hoc* explanation. The first term refers to a model able to fit a relationship between input and output, though simple enough to be understood. These models are built in order to be explainable, forcing many coefficients to zero and selecting a subset of features. Usually, these models are simple ML algorithms and are not widely used in DL.^86^ On the contrary, a *post hoc* explanation refers to attempting an explanation after the end of the training of the model. The second distinction is between a model-specific and a model-agnostic explanation. A model-specific explanation is limited to some classes of models and cannot be applied to others, while a model-agnostic explanation can be applied to different types of models. Of note, a model-based explanation is by definition model-specific; however, a model-specific explanation is not necessarily model-based.^91^ The third distinction is between a global and local explanation. A global explanation aims to provide general relationships learned by the model. In other words, it explains how much features contribute to the output across the entire dataset. On the other hand, a local explanation provides an explanation of a single input. In other words, it explains why a feature contributes to the risk of outcome for a single patient.^91,92^

#### Explainable AI for medical image analysis

##### Saliency mapping

Saliency mapping, also called visual explanation, is the most common form of explainable AI in medical imaging. Saliency maps use different techniques to show the important parts of an image for a decision. The most used techniques for saliency mapping in cardiovascular images are based on guided backpropagation, class activation mappings (CAMs), and Shapley additive explanation (SHAP). The first highlights pixels with the highest impact on the output, provides a local explanation, and can be model-specific (only for CNNs) or *post hoc*.^93,94^ CAMs are usually used in ensembles of CNNs as they replace the fully connected layers at the end of a CNN in order to create feature maps.^95^ These techniques provide local, model-specific, and *post hoc* explanations. Variants of CAMs (i.e. multi-scale CAM and gradient-weighted CAM) were used in CMR image analysis.^41,96^ SHAP is a model-agnostic approach that determines the marginal contribution of every feature to the model's output.^97,98^ In other words, it assigns a feature importance value to each element in a prediction, where a positive SHAP value indicates that a feature increases the probability of the prediction, while a negative value suggests a negative impact on the predicted probability. Applied to image analysis, in particular to CNN (Deep SHAP), it explains which part of the image has a positive contribution and which part has a negative contribution to the estimation.^42,98^

##### Textual explanation

This form of explainable AI adds a textual description to the model. There are three main types of textual explanation. The first one, called image captioning, uses LSTM trained with human-generated sentences for textual encoding.^99^ The second type is a variant of image captioning combined with visual explanation. In this way, a visual attention map is linked with a textual explanation. These models have been tested in other fields of medical image analysis with encouraging results, albeit not yet in cardiovascular imaging.^100,101^ The third type, called testing with concept activation vector (TCAV), trains the model as a *post hoc* analysis with a user-defined set of textual concepts and quantifies the sensitivity of this training model using concept activation vectors.^102^ TCAV was used in cardiovascular imaging analysis to identify biomarkers related to a cardiac disease detected from cine MRI.^103^

##### Example-based explanation

Example-based explanation is a method that uses examples to help understand why a model made a specific decision. Generally speaking, it is similar to how humans think by comparing new situations to past experiences. In particular, the model organizes information so that similar cases are grouped together, making it easier to find and use these comparisons.^91^ A modified version of the example-based explanation method was tested by Biffi *et al*.^104^ in CMR images to enhance differences in LV shape between hypertrophic cardiomyopathy and healthy controls.

## Case study and practical examples of deep learning prediction models

In the following paragraph, a case study illustrates how to plan a research project aimed at developing a DL prediction model using CMR images. Then, an overview of the application of DL prognostic prediction models in the field of arrhythmic risk prediction is provided.

### Case study

**Figure 2:**
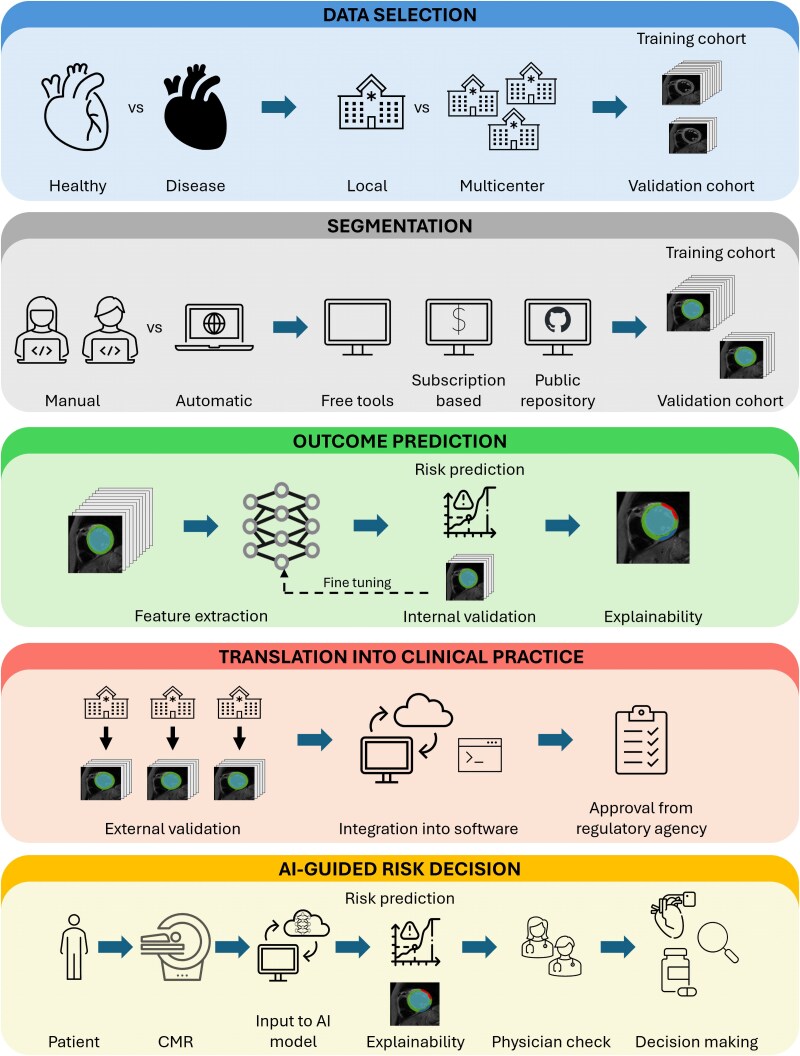
Case study. *(Top panel)* Dataset selection is critical to ensuring the model's validity and generalizability. Depending on the clinical question, cohorts of healthy individuals or patients with specific diseases are selected from local datasets or multicentre collaborations. For this purpose, biobank-scale non-invasive imaging datasets provide large, high-quality data resources. *(Second top panel)* Cardiac segmentation can be performed using manual, semi-automatic, or fully automatic approaches, depending on dataset size, project timeline, and budget. Freely available or subscription-based software tools offer user-friendly platforms with limited flexibility. Alternatively, deep learning segmentation models from public repositories provide greater performance and customization but typically require retraining on local datasets. *(Middle panel)* Feature extraction enables the model to incorporate imaging data and identify complex features for predicting patient-specific risk curves. No ready-to-use software currently exists for this task; instead, researchers must adapt and retrain code from public repositories, which demands programming expertise. Model performance should be validated on a separate cohort, and explainability must be ensured to confirm that the model focuses on clinically meaningful regions of interest. *(Penultimate panel)* After internal validation confirms the model's performance, external validation using heterogeneous cohorts is necessary. To enable clinical application, the model must be integrated into clinical software and approved by regulatory agencies. *(Bottom panel)* A possible clinical use of an AI-guided prediction model: following cardiac magnetic resonance imaging, the images are input into a pretrained AI model that generates a patient-specific outcome prediction along with an explanation. Clinicians assess the validity and interpretability of the result before determining the appropriate clinical strategy. *Neural network, medical device, and risk curves designed by Freepik* (www.freepik.com).

The case study is summarized in *Figure 2* and follows all the steps of the proposed pipeline.

The first step—selecting appropriate input data—is critical to ensuring the model's validity and generalizability. Biobank-scale non-invasive imaging offers an unprecedented opportunity to train and validate DL models on large, high-quality datasets. Several initiatives—such as the UK Biobank,^105^ Multi-Ethnic Study of Atherosclerosis (MESA),^106^ Automated Cardiac Diagnosis Challenge (ACDC),^84^ and the German National Cohort^107^—have released population-based CMR datasets that are readily accessible. The choice of dataset should align with the research question: the UK Biobank is ideal for population-level modelling, while ACDC and MESA provide disease-specific data suited to targeted applications. Matching the dataset characteristics to the model's purpose is essential for building robust and generalizable tools.

Multiple options are available for cardiac segmentation, where AI-based automatic segmentation tools with pretrained models can be used directly by uploading DICOM files. These tools—whether free (e.g. IntelliCardiac^108^ and SlicerHeart^109^) or subscription-based (e.g. cvi42^110^ and Medviso Segment CMR^111^)—are generally more user-friendly but offer limited flexibility. In contrast, freely available DL models hosted on public repositories (like GitHub) typically offer greater customizability and performance but are less accessible to non-technical users, requiring manual pre- and post-processing adjustments and retraining on local data to ensure optimal performance. Among them, the segmentation model developed by Bai *et al*.^64^ has been trained on one of the largest cohorts and demonstrated excellent performance.

The third step in the proposed pipeline is feature extraction, aimed at identifying imaging patterns relevant to predictive tasks. This can be accomplished using user-friendly tools such as *3D Slicer*^112^ or *Radiomics*.*org*,^113^ which allow radiomic feature extraction through graphical interfaces without the need for coding. Alternatively, DL-based feature extraction offers greater flexibility and can capture complex features directly from images. However, no ready-to-use software exists for this purpose, and researchers must adapt code from public repositories, requiring programming skills and retraining on local datasets.

The fourth step proposed is explainability, which is essential for interpreting model outputs and fostering trust in clinical applications. While explainability tools are available for traditional ML, those tailored for DL in medical imaging remain limited. Some user-friendly platforms such as *Zegami*,^114^  *Google Cloud AutoML*,^115^  *IBM Watson Studio*,^116^  *Microsoft Azure Machine Learning*,^117^ and *KNIME*^118^ offer basic visual explanations, including saliency maps or feature attribution. These platforms are useful for exploratory research but are not designed for complex imaging tasks like outcome prediction. In contrast, advanced explainability methods, as described above, can provide deeper insights into DL model predictions. However, these tools require manual implementation using code adapted from public repositories, as no off-the-shelf solutions currently exist for CMR-specific explainability.

### Deep learning for arrhythmic risk prediction in cardiomyopathies

The ability to analyse a wide range of heterogeneous data has strengthened the conviction that AI may have a radical impact on the arrhythmic risk stratification of patients with cardiomyopathies.^4^ Currently, clinical criteria for implantable cardioverter defibrillator (ICD) candidacy as primary prevention are largely based on regression models that consider left ventricular ejection fraction, the presence of particular features (e.g. myocardial fibrosis), or genetic mutations.^119,120^ CMR plays a role in guiding ICD implantation when LGE is detected, although no clear consensus exists regarding its extent and location.^4,119,121^ To date, various regression-based risk scores—Atherosclerosis Risk in Communities (ARIC) score,^122^ Seattle proportional risk model,^123^ VFRisk,^124^ MADIT-ICD^125^—have been proposed, although none have demonstrated sufficient performance in external validation to be adopted in clinical practice.^120^

To address these limitations, previous studies have explored the use of ML algorithms to predict the risk of SCD in different cardiomyopathies, as presented by Barker *et al*.^126^ in a recent systematic review. They identified six studies comparing ML models with Cox regression models for SCD prediction. Five of the six ML models outperformed regression models, with an area under the curve (AUC) ranging from 0.71 to 0.96.^127–132^ However, these findings were met with criticism. In fact, only one model was tested in a validation cohort, raising concerns that the high accuracy reported by the others reflected overfitting. Furthermore, when compared with Cox regression models, the overall improvement in performance was marginal.^126^ Additionally, only two studies volunteered the capacity to share data, and no studies shared access to the code used in their ML analysis.

AI tools have also been adopted to directly extract features from ECG signals to predict arrhythmic outcomes. In this regard, Kolk *et al*.^133^ developed a multimodal ML model using ECG time-series to predict 3-year non-arrhythmic mortality, achieving an AUC 0.79 in external validation. Rogers *et al*.^134^ implemented a model to predict SCD equivalents using monophasic action potential data from a cohort of 42 patients. DL models trained on different types of electric signals data (ECG, 24 h ECG, intracardiac electrograms) have also been tested.^135–137^ While promising, these models have been criticized for being developed on *ad hoc* datasets with small sample sizes and lacking external validation.^138^

**Figure 3:**
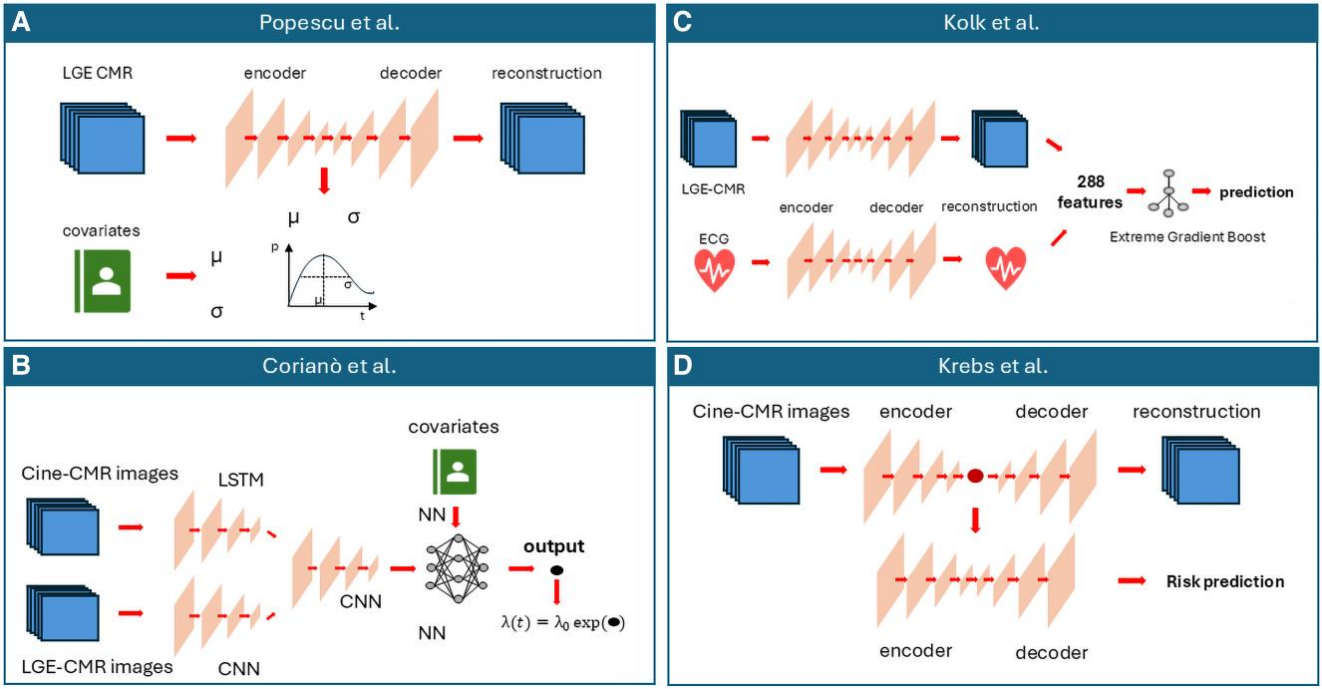
State-of-the-art deep learning models for predicting arrhythmic risk from cardiac magnetic resonance images. *A*) The model developed by Popescu *et al*.^41^ consists of two subnetworks that process LGE images and covariates as input data. A 3D convolutional encoder–decoder extracts features from the image branch and predicts the location and scale parameters (σ, μ) that characterize the probability distribution of the patient's time to a major arrhythmic event. *B*) The model proposed by our group uses cine and LGE images, along with clinical covariates, as input variables. Cine images are processed by convolutional long short-term memory networks, while the LGE branch uses convolutional neural networks. These two branches are flattened and merged with the covariate branch. The resulting vector is processed by a multilayer neural network, returning an output that is used as the exponent of the Cox hazard function. *C*) Kolk *et al*.^113^'s model consists of two branches that process LGE and ECG data as input. Both branches utilize a variational autoencoder with convolutional layers for feature extraction. These features are then analysed by an Extreme Gradient Boosting algorithm to predict arrhythmic risk. *D*) Krebs *et al*.^60^ built a model consisting of an autoencoder that processes short- and long-axis cine images. The extracted features are used as input for a variational autoencoder for risk stratification.

Advancements in cardiac imaging techniques have opened new possibilities for risk prediction. Early studies applied AI to characterize proarrhythmogenic substrates from CMR images. For instance, Okada *et al*.^139^ used AI models to detect tissue irregularities in transition zones to predict arrhythmias, while Balaban *et al*.^140^ derived a LV shape score from 3D reconstructions. More recently, multi-input DL models have been developed to analyse CMR images alongside other types of data to predict outcomes. At the current state-of-the-art, four types of multi-input DL architectures have been applied to arrhythmic risk prediction in cardiomyopathies,^41,42,59,60^ as summarized in *Figure 3*. In 2022, Popescu *et al*.^41^ demonstrated that combining clinical covariates with LGE images in a unified DL model can accurately predict major arrhythmic events in patients with ischaemic dilated cardiomyopathy. They compared four prognostic prediction models: regression-based, DL with clinical covariates only, DL with images only, and DL with both covariates and images. Their findings highlighted that a DL approach outperforms regression models and that combining clinical and image data enables the model to learn novel features. The model proposed by the authors consisted of two subnetworks operating on LGE-CMR images and clinical covariate data, respectively (*Figure 3A*). Cardiac segmentation was performed using a custom U-Net,^43^ and features were extracted via a 3D convolutional encoder–decoder used for both survival analysis and image reconstruction.^41^ The encoded features were categorized into risk groups and passed through a dense layer to predict each patient's location and scale parameters, which characterized the probability distribution of logarithmic time to a major arrhythmic event. Explainability was provided by adopting a gradient-weighted CAM on the CMR subnetwork. The model was internally validated in a cohort of 156 patients and externally validated in a cohort of 113 patients, achieving concordance indexes of 0.83 and 0.74, respectively.

Our group developed a multi-input deep neural regression network composed of three main branches operating on cine, LGE, and covariates (*Figure 3B*).^59^ The cine-CMR branch consisted of convolutional LSTM, while the LGE-CMR branch used CNNs. These branches produced a multidimensional array that was flattened and concatenated with the clinical data array. The final array was processed through a novel DL survival model comprising multiple layers, with the output used as a coefficient in the Cox hazard function. This model was trained and tested in a cohort of 108 and 46 patients, respectively, achieving an AUC of 0.64 at 3 years in the test set. Kolk *et al*.^42^ designed a model with two branches for LGE-CMR and ECG inputs (*Figure 3C*). The extracted features were used to train a supervised ML model via extreme gradient boosting to predict the 1-year risk of malignant ventricular arrhythmia. A two-step explainability approach was applied: SHAP analysis for feature importance and gradient-based attention mapping to generate patient-specific heatmaps highlighting key MRI regions. The model was trained and tested on cohorts of 186 and 103 patients, respectively, achieving an AUC of 0.84 for predicting ICD intervention within 1 year.

Lastly, Krebs *et al*.^60^ trained an autoencoder NN on short- and long-axis cine images for feature extraction (*Figure 3D*). The model output was used as input to a variational autoencoder that generated a survival risk score. This model was only evaluated on its training cohort, and the authors called for external validation by other research groups. This model was only evaluated on its training cohort, and the authors called for external validation by other research groups.

All four models presented are technically innovative and demonstrate promising predictive performance. However, they were tested on relatively small datasets, which limits their generalisability. Further validation in larger and more diverse cohorts is essential. Moreover, none of these models is currently available or ready for clinical use. While most authors have shared the code necessary to reproduce the pipelines, none of the pretrained models have been released or commercialized for clinical application. Therefore, although DL paves the way for personalized risk prediction, the field remains confined to research settings.

## Conclusions

DL promises a high level of accuracy in risk prediction, and its use is spreading to fill different gaps of knowledge in cardiovascular science. An exciting opportunity in this field lies in the possibility of analysing cardiovascular images, particularly CMR images. Nonetheless, building an accurate and reliable DL model requires a strict methodological approach, starting from a conceptualization of the clinical problem, passing through a correct choice of the variables to analyse and the model's architecture, and concluding with the provision of an explanation of the model's prediction.

## Lead author biography


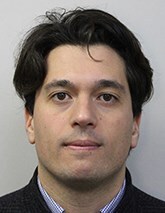


Mattia Corianò is a cardiologist and a PhD student at the University of Padua. He has recently joined the Computational Cardiac Imaging group at the Medical Research Council Laboratory of Medical Science—Imperial College London. His clinical interests include heart failure, CMR, and heart transplantation. His research focuses on the application of AI in cardiology.

## Data Availability

No new data were generated or analysed in support of this research.
